# Analytic estimation of transition between instantaneous eigenstates of quantum two-level system

**DOI:** 10.1038/s41598-018-35741-5

**Published:** 2018-11-27

**Authors:** Takayuki Suzuki, Hiromichi Nakazato, Roberto Grimaudo, Antonino Messina

**Affiliations:** 1 Department of Physics, Waseda University, Tokyo, 169-8555 Japan; 2 Dipartimento di Fisica e Chimica dell’Universitá di Palermo, Via Archirafi, 36, I-90123 Palermo, Italy; 3 INFN, Sezione di Catania, Catania, Italy; 4 Dipartimento di Matematica ed Informatica dell’Universita di Palermo, Via Archirafi 34, I-90123 Palermo, Italy

## Abstract

Transition amplitudes between instantaneous eigenstates of a quantum two-level system are evaluated analytically on the basis of a new parametrization of its evolution operator, which has recently been proposed to construct exact solutions. In particular, the condition under which the transitions are suppressed is examined analytically. It is shown that the analytic expression of the transition amplitude enables us, not only to confirm the adiabatic theorem, but also to derive the necessary and sufficient condition for quantum two-level system to remain in one of the instantaneous eigenstates.

## Introduction

A quantum system described by a time-dependent nondegenerate Hamiltonian *H*(*t*), such that $[H(t),\,H(t^{\prime} )]\ne 0$, does not possess stationary states, since, in general, the mean value of an observable changes in time. The eigenstates of *H*(*t*) (instantaneous eigenstates) depend on time and evolve into those states that are no longer the corresponding eigenstates of the Hamiltonian at that time instant. The deviations from the eigenstates, however, become negligible under the situation where the so-called adiabatic theorem^1–3^ is applicable. The theorem states that the *n*th instantaneous eigenstate evolves remaining with continuity in the *n*th eigenstate at any time instant. The condition for such an occurrence in a somewhat intuitive sense is that the quantum dynamics of the system is governed by a Hamiltonian that changes vanishingly slowly in time, yielding a finite variation over an infinite time interval *T* → ∞ (adiabatic limit).

The adiabatic theorem plays an important role and constitutes a basis in a variety of research fields^4–9^. In its proofs, the transitions to other instantaneous eigenstates over a finite time interval *T* are estimated to be suppressed by 1/*T* ^2^. The adiabatic theorem holds irrespectively of details of the system under consideration, thus making it applicable to a wide class of quantum systems. On the other hand, any physically realizable process can not be in the adiabatic limit because the time duration *T* can not be made infinite and the physical process can be considered at most approximately adiabatic. It is therefore of practical relevance to understand how well it is approximated as adiabatic and to estimate what the possible deviations from the adiabatic limit are. Notice that we are mainly concerned about the adiabaticity of quantum state, that is, whether the quantum system remains in one of the instantaneous eigenstates for all times, when the time evolution can only be approximately adiabatic. There are classical papers on these issues^10,11^, but the so-called Marzlin-Sanders inconsistency found in this century^12^ has provoked heated discussions^13–26^.

Notice that the theorem gives little information on these issues and a precise estimation of such deviations would require a knowledge of the exact dynamics. It is thus desirable to have an access to exact solutions of the dynamics and then natural to expect to have new insights on these issues once a new strategy to construct analytical solutions has been proposed. In this respect, it is worth while to stress that such a strategy for obtaining exact solutions for quantum two-level systems was proposed recently^27–29^ and actually several exactly solvable examples, exploitable also for interacting qudits, have newly been obtained^30–34^ according to the strategy^29^.

The purpose of this paper is twofold. First, the transition amplitudes between instantaneous eigenstates of the general time-dependent Hamiltonian for quantum two-level system are expressed analytically in terms of appropriate quantities that parametrize the dynamics. Second, the necessary and sufficient condition under which the transition from one of the instantaneous eigenstates to the other one is suppressed is derived and discussed. Two solvable examples that can represent adiabatic processes are added to illustrate how such transition amplitudes behave as the time interval *T* becomes large, confirming the adiabatic theorem and the validity of the condition found here.

## Transition Amplitude Between Instantaneous Eigenstates

Let a quantum two-level system be described by a time-dependent Hamiltonian

1 $$H(t)=(\begin{array}{cc}{\rm{\Omega }}(t) & \omega (t)\\ {\omega }^{\ast }(t) & -{\rm{\Omega }}(t)\end{array}),\,\omega (t)=|\omega (t)|{e}^{i{\varphi }_{\omega }(t)},$$

where Ω and *ω* are time-dependent real and complex functions, respectively. The instantaneous eigenvalues ${E}_{\pm }(t)=\pm \sqrt{{{\rm{\Omega }}}^{2}(t)+{|\omega (t)|}^{2}}$ and eingenstates ${|\pm \rangle }_{t}$,

2 $${|+\rangle }_{t}=(\begin{array}{c}{e}^{\frac{i}{2}{\varphi }_{\omega }}\cos \,\frac{\theta }{2}\\ {e}^{-\frac{i}{2}{\varphi }_{\omega }}\sin \,\frac{\theta }{2}\end{array}),\,{|-\rangle }_{t}=(\begin{array}{c}{e}^{\frac{i}{2}{\varphi }_{\omega }}\sin \,\frac{\theta }{2}\\ -\,{e}^{-\frac{i}{2}{\varphi }_{\omega }}\cos \,\frac{\theta }{2}\end{array}),$$

are both time dependent, where $\tan \,\theta =|\omega |/{\rm{\Omega }}$. Here and in the following *t*-dependence will not be explicitly written and all quantities are understood to be time dependent unless otherwise stated explicitly.

According to the strategy proposed in^29^, the evolution operator *U*(*t*) is given by

3 $$U=(\begin{array}{cc}a & b\\ -{b}^{\ast } & {a}^{\ast }\end{array}),\,a=\,\cos \,\chi {e}^{-\frac{i}{2}({\rm{\Theta }}-{\varphi }_{\omega }+\varphi +{\varphi }_{\omega }(0))},\,b=-\,i\,\sin \,\chi {e}^{-\frac{i}{2}({\rm{\Theta }}-{\varphi }_{\omega }-\varphi -{\varphi }_{\omega }(0))},$$

where Θ is an arbitrary real-valued function of time with Θ(0) = 0 and

4 $$\chi ={\int }_{0}^{t}\,dt^{\prime} \frac{|\omega (t^{\prime} )|}{\hslash }\,\cos \,{\rm{\Theta }}(t^{\prime} ),$$

5 $$\varphi ={\int }_{0}^{t}\,dt^{\prime} \frac{\mathrm{2|}\omega (t^{\prime} )|}{\hslash }\,\frac{\sin \,{\rm{\Theta }}(t^{\prime} )}{\sin \,2\chi (t^{\prime} )}\mathrm{.}$$

The function Ω, representing the longitudinal magnetic field in the case of spin 1/2, is specified by

6 $${\rm{\Omega }}=\frac{\hslash }{2}(\dot{{\rm{\Theta }}}-{\dot{\varphi }}_{\omega })+|\omega |\,\sin \,{\rm{\Theta }}\,\cot \,2\chi ,$$

where dot (^·^) stands for derivative w.r.t. time. The original idea of ^29^ is such that, if Ω is so adjusted to satisfy (6) for an arbitrarily given *ω* and a parameter function Θ, then *U* is explicitly given in terms of them as in (3). One may, however, regard (3) as a *new parametrization* of *U*. In fact, if the evolution operator *U* is so parametrized by *χ*, Θ and *ϕ* as in (3), the Eqs (4–6) are enough to make *U* satisfy the Schrödinger equation. The three parameters *χ*, Θ and *ϕ* connect, though implicitly in general, the three parameters in the Hamiltonian Ω and $\omega =|\omega |{e}^{i{\varphi }_{\omega }}$ and those in the evolution operator *a* and *b* (|*a*|^2^ + |*b*^2^| = 1). It is stressed that this is nothing but a representation of the Schrödinger equation and nothing has been imposed on the character of Ω and *ω*. It is not difficult to understand the meanings of the parameters introduced above. The parameter *χ* parametrizes the transition “angle,” while the others Θ and *ϕ* their “phases,” see Eq. (3).

It is convenient for the later use to rewrite the evolution operator *U* in a compact form

7 $$U=(\begin{array}{cc}\cos \,\chi {e}^{-\frac{i}{2}({\rm{\Theta }}+\varphi -{\varphi }_{\omega }+{\varphi }_{\omega }(0))} & -i\,\sin \,\chi {e}^{-\frac{i}{2}({\rm{\Theta }}-\varphi -{\varphi }_{\omega }-{\varphi }_{\omega }(0))}\\ -i\,\sin \,\chi {e}^{\frac{i}{2}({\rm{\Theta }}-\varphi -{\varphi }_{\omega }-{\varphi }_{\omega }(0))} & \cos \,\chi {e}^{\frac{i}{2}({\rm{\Theta }}+\varphi -{\varphi }_{\omega }+{\varphi }_{\omega }(0))}\end{array})={e}^{\frac{i}{2}{\sigma }_{z}{\varphi }_{\omega }}{e}^{-i{\rm{\Phi }}{\boldsymbol{n}}\cdot {\boldsymbol{\sigma }}}{e}^{-\frac{i}{2}{\sigma }_{z}{\varphi }_{\omega }(0)},$$

where $${\boldsymbol{\sigma }}=({\sigma }_{x},\,{\sigma }_{y},\,{\sigma }_{z})$$ is the Pauli matrix vector. We have introduced a unit vector $${\boldsymbol{n}}=({n}_{x},\,{n}_{y},\,{n}_{z})$$ with $${n}_{x}=\,$$$$\sin \,\xi \,\cos \,{\phi }_{-}=\frac{\sin \,\chi }{\sin \,{\rm{\Phi }}}\,\cos \,{\phi }_{-}$$, $${n}_{y}=\,\sin \,\xi \,\sin \,{\phi }_{-}=\frac{\sin \,\chi }{\sin \,{\rm{\Phi }}}\,\sin \,{\phi }_{-}$$ and $${n}_{z}=\,\cos \,\xi =\frac{\cos \,\chi }{\sin \,{\rm{\Phi }}}\,\sin \,{\phi }_{+}$$, and $$\cos \,{\rm{\Phi }}=\,\cos \,\chi \,\cos \,{\phi }_{+}$$, where angles are defined as $${\phi }_{\pm }=\frac{1}{2}({\rm{\Theta }}\pm \varphi )$$. Then it is straightforward to calculate transition amplitudes between instantaneous eigenstates. For example, the transition from the initial state $$|+{\rangle }_{0}$$ to the other eigenstate $$|-{\rangle }_{t}$$ occurs with an amplitude ($${\theta }_{0}=\theta \mathrm{(0)}$$)

8 $${}_{t}\langle \,-\,|U(t)|\,+\,{\rangle }_{0}=\,\cos \,\chi \,\cos \,{\phi }_{+}\,\sin \,\frac{\theta -{\theta }_{0}}{2}-\,\sin \,\chi \,\sin \,{\phi }_{-}\,\cos \,\frac{\theta -{\theta }_{0}}{2}+i(\sin \,\chi \,\cos \,{\phi }_{-}\,\cos \,\frac{\theta +{\theta }_{0}}{2}-\,\cos \,\chi \,\sin \,{\phi }_{+}\,\sin \,\frac{\theta +{\theta }_{0}}{2}).$$

Similarly, the other amplitudes are concisely expressed in terms of trigonometric functions of *χ*, ${\phi }_{\pm }$ and *θ*^35^. Notice that these expressions are exact and no approximation is involved.

## Necessary and Sufficient Condition for Staying in One of the Instantaneous Eigenstates at All Times

The analytical expression of transition amplitude (8) enables us to investigate the condition under which no transition between different eigenstates at any time is allowed, ${}_{t}\langle -|U(t)|+\rangle _{0}=\mathrm{0,}\,\,\forall t\ge 0$. The condition requires the following relations to hold

9 $$\tan \,\frac{\theta +{\theta }_{0}}{2}=\,\tan \,\chi \frac{\cos \,{\phi }_{-}}{\sin \,{\phi }_{+}},\,\tan \,\frac{\theta -{\theta }_{0}}{2}=\,\tan \,\chi \frac{\sin \,{\phi }_{-}}{\cos \,{\phi }_{+}}.$$

They are combined to yield

10 $$\tan \,\theta =\frac{2\,\tan \,\chi \,\cos \,\varphi }{\sin \,{\rm{\Theta }}\,\cos \,\varphi \mathrm{(1}-{\tan }^{2}\chi )+\,\cos \,{\rm{\Theta }}\,\sin \,\varphi \mathrm{(1}+{\tan }^{2}\chi )}=\frac{|\omega |}{\hslash }\frac{1}{y\,\cot \,2\chi +\frac{\dot{\chi }}{\sin \,2\chi }\,\tan \,\varphi },$$

where *y* is defined by

11 $$y=\frac{|\omega |}{\hslash }\,\sin \,{\rm{\Theta }}.$$

Notice that the quantities in the Hamiltonian are then expressed, in terms of *y*, as

12 $$|\omega |=\hslash \sqrt{{y}^{2}+{\dot{\chi }}^{2}},\,{\rm{\Omega }}=\frac{\hslash }{2}(\dot{{\rm{\Theta }}}-{\dot{\varphi }}_{\omega })+\hslash y\,\cot \,2\chi ,$$

while

13 $$\tan \,{\rm{\Theta }}=\frac{y}{\dot{\chi }},\,\dot{\varphi }=\frac{2y}{\sin \,2\chi }.$$

We obtain, from Eq. (10),

14 $${\dot{\varphi }}_{\omega }=\dot{{\rm{\Theta }}}-\frac{2\dot{\chi }}{\sin \,2\chi }\,\tan \,\varphi \mathrm{.}$$

Equations (9) also yield

15 $$\tan \,{\theta }_{0}=\frac{2\,\tan \,\chi }{\tan \,{\rm{\Theta }}\,\cos \,\varphi \mathrm{(1}+{\tan }^{2}\chi )+\,\sin \,\varphi \mathrm{(1}-{\tan }^{2}\chi )},$$

which can be reduced to the following differential equation (assuming nonvanishing $\tan \,{\theta }_{0}\ne 0$ without loss of generality)

16 $$\frac{d}{dt}[\cos \,\chi (\sin \,\chi \,\sin \,\varphi +\frac{\cos \,\chi }{\tan \,{\theta }_{0}})]=0.$$

The solution is easily found to be $\sin \,\varphi =\frac{\tan \,\chi }{\tan \,{\theta }_{0}}$, which leads to *θ* = *θ*_0_ and ${\dot{\varphi }}_{\omega }=0$. Actually, the differentiation of the solution w.r.t. *t* yields $y=\frac{\dot{\chi }\,\tan \,\chi }{\cos \,\varphi \,\tan \,{\theta }_{0}}$, resulting in $|\omega |=\frac{\hslash \dot{\chi }}{\cos \,\varphi }$ and ${\rm{\Omega }}=\frac{\hslash \dot{\chi }}{\cos \,\varphi \,\tan \,{\theta }_{0}}$. The result is consistent but trivial in the sense that the instantaneous eigenstates do not at all evolve in time.

In order to allow them to evolve, we need to admit to add a finite quantity which is not constant but whose velocity can be neglected. (This concept is in accord with the notion of limit of adiabaticity, i.e., a finite change with an infinitesimal velocity). Let us introduce such a parameter *c* and equate the quantity in the square parentheses in (16) with $\frac{1+c}{\tan \,{\theta }_{0}}$. Then the solution would be parametrized as

17 $$\sin \,\varphi =\frac{\tan \,\chi }{\tan \,{\theta }_{0}}(1+\frac{c}{{\sin }^{2}\chi }).$$

The function *c* (*c*(0) = 0) is assumed to be almost constant in time but takes a finite nonvanishing value at *t* > 0, like in the case where *c* is a function of *t*/*T* (0 ≤ *t* ≤ *T*). We will neglect terms proportional to $\dot{c}\propto \mathrm{1/}T$, but keep those proportional to $\dot{\chi }$ or *c*. Differentiation of (17) w.r.t. *t* gives

18 $$y=\frac{\dot{\chi }\,\tan \,\chi }{\cos \,\varphi \,\tan \,{\theta }_{0}}(1-({\cot }^{2}\chi -1)c),$$

which yields (see (12) and (14))

19 $${\rm{\Omega }}=\hslash \frac{\dot{\chi }}{\cos \,\varphi }\frac{1+2c}{\tan \,{\theta }_{0}},$$

20 $$\omega =\hslash \frac{\dot{\chi }}{\cos \,\varphi }\sqrt{1-\frac{4c(1+c)}{{\tan }^{2}{\theta }_{0}}}{e}^{i{\varphi }_{\omega }},\,{\dot{\varphi }}_{\omega }\sim 0.$$

The consistency condition $\tan \,\theta =|\omega |/{\rm{\Omega }}$ requires that

21 $$c=\frac{1}{2}(\frac{\cos \,\theta }{\cos \,{\theta }_{0}}-1).$$

Thus we have found that the condition that no transition between different eigenstates occurs requires that the Hamiltonian has to be parametrized as above with arbitrary functions $\chi ,{\varphi }_{\omega }$ and *c*. The condition $|\dot{\chi }|\gg |\dot{c}| \sim 0$ is equivalent to $\dot{\theta } \sim 0$, implying, together with ${\varphi }_{\omega } \sim 0$, that the instantaneous eigenstates have to vary infinitesimally slowly in time (*necessity*), though the Hamiltonian itself can vary at a finite rate^26^. It is interesting to see that Eq. (19) can actually be integrated, thus assuring that such a parametrization is always possible, to yield

22 $$\tan \,\varphi =\frac{\cos \,{\theta }_{0}}{\sin (2\zeta )}(\frac{\tan \,{\theta }_{0}}{\tan \,\theta }-\,\cos (2\zeta )),$$

where $\zeta =\frac{1}{\hslash }{\int }_{0}^{t}\,ds\sqrt{{{\rm{\Omega }}}^{2}(s)+|\omega (s{)|}^{2}}$. This, together with (17), results in the specification of *χ* in terms of Ω and |*ω*|.

It is also possible to show that when the Hamiltonian is characterized by the above Ω and *ω*, (19) and (20), the transition amplitude (8) vanishes, provided $\dot{c} \sim 0$, i.e., $\dot{\theta } \sim 0$ (*sufficiency*). (In order to keep the relation (17) intact, we would need a correction term proportional to $\dot{c}$ in *y*, $\dot{c}{(\cos \varphi \tan {\theta }_{0})}^{-1}$, which was neglected in (18)).

### Examples

As an example, we may consider a particular case of physical interest. Choose the functions *χ* and *y* as

23 $$\tan \,\chi =\,\sin \,\alpha t,\,y={\nu }_{0}\,\sin \,\alpha t$$

with parameters ${\nu }_{0} > 0$ and $\alpha \propto \mathrm{1/}T$. Then we have

24 $$|\omega |=\hslash \sqrt{{\nu }_{0}^{2}{\sin }^{2}\alpha t+{(\frac{\alpha \cos \alpha t}{1+{\sin }^{2}\alpha t})}^{2}},$$

25 $${\rm{\Omega }}=\frac{\hslash }{2}(\dot{{\rm{\Theta }}}-{\dot{\varphi }}_{\omega })+\frac{\hslash {\nu }_{0}}{2}{\cos }^{2}\alpha t,$$

where $\tan \,{\rm{\Theta }}=\frac{{\nu }_{0}}{\alpha }\mathrm{(1}+{\sin }^{2}\alpha t)\tan \,\alpha t$. Observe that the physical system under consideration could be well approximated for small $\alpha ,\,{\dot{\varphi }}_{\omega } \sim 0$ by

26 $$|\omega |\sim \hslash {\nu }_{0}|\sin \,\alpha t|,\,{\rm{\Omega }}\sim \frac{\hslash {\nu }_{0}}{2}{\cos }^{2}\alpha t$$

except for the initial transient times, where we have

27 $$|\omega (0)|=\hslash \alpha ,\,{\rm{\Omega }}(0)=\hslash {\nu }_{0}.$$

By setting $\alpha T=\frac{\pi }{2}$, we are going to consider a physical process where the magnetic field (for spin $\frac{1}{2}$ case), starting from an approximately longitudinal one (27) if $\alpha =\frac{\pi }{2T}\ll {\nu }_{0}$, varies slowly in time characterized by the trigonometric functions (26), reaching an approximately transversal one

28 $$|\omega (T)|=\hslash {\nu }_{0},\,{\rm{\Omega }}(T)=\frac{\hslash }{2}(\frac{{\alpha }^{2}}{2{\nu }_{0}}-{\dot{\varphi }}_{\omega }(T))\ll \hslash {\nu }_{0}.$$

Clearly the adiabaticity of the process is characterized by the inequalities

29 $$\frac{\alpha }{{\nu }_{0}}=\frac{\pi }{2T{\nu }_{0}}\ll 1,\,\frac{{\dot{\varphi }}_{\omega }}{{\nu }_{0}}\ll 1.$$

**Figure 1:**
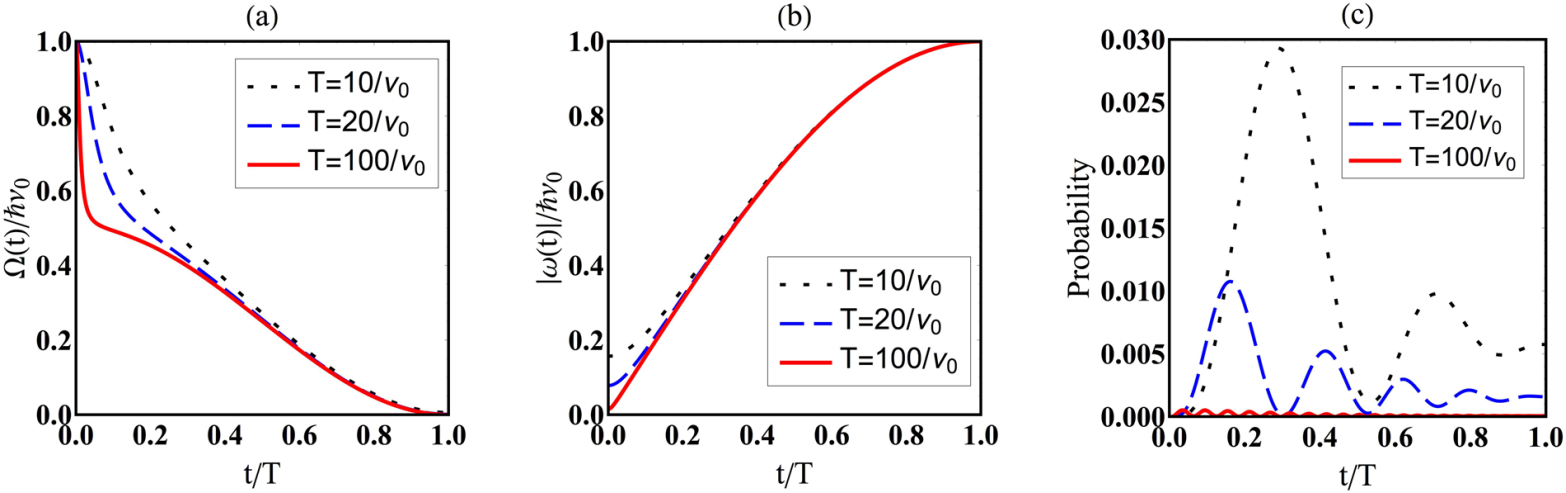
(**a**) Magnetic fields Ω in (25), (**b**) |*ω*| in (24) and (**c**) transition probability ${|}_{t}{\langle -|U(t)|+\rangle }_{0}{|}^{2}$ as functions of *t* for *ν*_0_*T* = 10 (dotted black lines), 20 (broken blue lines) and 100 (solid red lines).

Observe that in this case the parameter *ν*_0_ can be viewed as a characteristic frequency relevant to the energy of the system, while *α* is the fundamental frequency governing the variation of the Hamiltonian. Since we are given an analytical expression for every quantity (for example, $\varphi (t)=\frac{{\nu }_{0}}{4\alpha }\mathrm{(6}\alpha t-\,\sin \,2\alpha t)$), the transition amplitudes and probabilities are calculated without any approximation. The figures in Fig. 1 show the behaviour of transition probability ${|}_{t}{\langle -|U(t)|+\rangle }_{0}{|}^{2}$ as a function of time *t* for several values of ${\nu }_{0}T$, together with that of longitudinal (Ω) and transversal (|*ω*|) magnetic fields. It is evident that in the large ${\nu }_{0}T\gg 1$ limit, the transition probability becomes vanishingly small.

**Figure 2:**
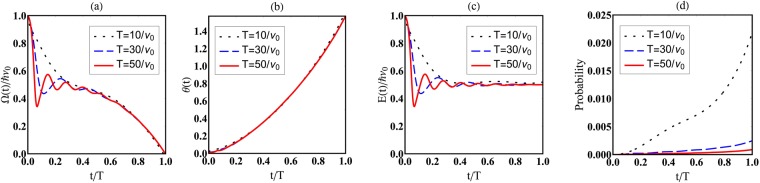
A Hamiltonian that keeps oscillating even at large *ν*_0_*T* can suppress the transition between different instantaneous eigenstates provided the latters change infinitesimally slowly in the limit. Panels (**a**) and (**c**) show that the longitudinal magnetic field and the instantaneous eigenvalue oscillate even for large values of *ν*_0_*T*, while the corresponding eigenstates characterized by the angle *θ* varies slowly as a function of time *t* for larger values of *ν*_0_*T* (**b**) and the transition probability becomes vanishingly small (**d**), realizing an adiabatic process.

In the above example, the Hamiltonian itself varies infinitesimally slowly, realizing a process where the instantaneous eigenstates do not make transitions. We may consider another example that realizes the same situation even if the Hamiltonian varies with a nonvanishing rate. For example, we may choose such a *χ* that realizes $\varphi ={\nu }_{0}t$ with a finite parameter *ν*_0_ and $c=-\,\frac{1}{2}(\frac{t}{T}{)}^{3}$ according to (17) and ${\varphi }_{\omega }=0$, which means that the speed of variation of *χ* is essentially *ν*_0_, a finite value irrelevant to *T*, while the other parameter *c* varies slowly in time with $\dot{c}$ proportional to 1/*T*. In this case, as Eqs (19) and (20) show, the characteristic frequency of the magnetic field (i.e., Hamiltonian) is *ν*_0_, while its instantaneous eigenstates become slowly varying for large *T*. The persistence of oscillations in Ω and *E* (Fig. 2(a,c)) even for large *T* and its absence in *θ* (Fig. 2(b)) are manifestations of these statements. The transition probability tends to vanish for large *T* (Fig. 2(d)) even if the Hamiltonian and its eigenvalues vary with finite speeds ((a) and (c)). This fact confirms that no transitions between instantaneous eigenstates are allowed for large *T* even for such a varying Hamiltonian. Our approach allows us to exhibit an example in which the system stays in its instantaneous eigenstate, even though the Hamiltonian varies with a nonvanishing rate.

## Summary and Discussions

In summary, the adiabatic theorem is confirmed on the basis of the explicit expressions of the transition amplitudes (see (8) and also refer to^35^), which are derived from the new parametrization of the evolution operator for the quantum two-level system^29^. What is stressed here is that these expressions enable us to evaluate such transition amplitudes directly in any physical situation, from the adiabatic to diabatic cases. Furthermore, we have made it clear that the necessary and sufficient condition that the state prepared initially in one of the eigenstates of the Hamiltonian remains in the corresponding instantaneous eigenstate at any time is that *the speed of variation of the eigenstate is negligible*, i.e.,

30 $$\dot{\theta }\sim 0,\,{\dot{\varphi }}_{\omega }\sim 0,$$

compared with the frequency (i.e., energy $/\hslash$) of the system.

Notice that this condition, though mentioned in^26^, has never been explicitly derived so far. It is different from the traditional one $\hslash {|}_{t}{\langle -|\dot{H}|+\rangle }_{t}|/({E}_{-}(t)-{E}_{+}(t{))}^{2}\ll 1$ and is free from the insufficiency found for the latter^12,13^. Actually the traditional condition is read in the case of two-level systems as $\hslash \sqrt{{(\frac{1}{2}\dot{\theta })}^{2}+{(\frac{1}{2}{\dot{\varphi }}_{\omega }\sin \theta )}^{2}}$ $\ll {E}_{+}-{E}_{-}=2\sqrt{{{\rm{\Omega }}}^{2}+|\omega {|}^{2}}$and therefore even if this condition is satisfied, a resonant transition with $\hslash |{\dot{\varphi }}_{\omega }|={E}_{+}-{E}_{-}\ne 0$ can occur between eigenstates when the magnetic field is almost longitudinal $0 < |\,\sin \,\theta |\ll 1$. Needless to say, such situations are not allowed according to the conditions (30) because one of them is not satisfied $\hslash |{\dot{\varphi }}_{\omega }|\ll /{E}_{+}-{E}_{-}$. It is remarked here that the conditions (30) actually follow from $\dot{\theta }\,\sin \,\theta \sim 0$ under the assumption of nonvanishing factor $\sin \,\theta \ne 0$. When this factor becomes vanishingly small at all times, no further conditions are required for other quantities^25^, but this is a trivial case where the magnetic field always points to the *z*-direction, a case described by a diagonal Hamiltonian. Notice also that there is an exceptional situation. If the Hamiltonian itself becomes vanishingly small ${\rm{\Omega }}\sim 0,\,|\omega |\sim 0$, the above condition will loose its meaning and our approach can say nothing about the adiabaticity of state^36^.

Remember that it is generally difficult to eliminate potential loopholes in the discussions on and proposals of the adiabatic condition because we are hardly able to reach the exact dynamics except for some limited cases, even for simple quantum two-level systems. It is stressed in this respect that our conclusion is derived from the analytic parametrization of the exact dynamics of the two-level system and offers a definite answer, with its range of validity specified, to this issue for the quantum two-level systems. Further applications to other fundamental issues, including, e.g., the Landau–Zener transition^4,5^, will be published elsewhere.

## Data Availability

All data generated or analysed during this study are included in this article.
